# Predictors, prevalence and prognostic role of pulmonary hypertension in patients with chronic kidney disease: a systematic review and meta-analysis

**DOI:** 10.1080/0886022X.2024.2368082

**Published:** 2024-06-28

**Authors:** Chunlong Lin, Qilong Ge, Lei Wang, Pan Zeng, Mingmin Huang, Dan Li

**Affiliations:** Department of Respiratory and Critical Care Medicine, Yueyang municipal Hospital of Hunan Normal University, Hunan, China

**Keywords:** Predictors, prevalence, prognostic, pulmonary hypertension, chronic kidney disease, meta-analysis

## Abstract

**Background:**

To estimate the predictors, prevalence and prognostic role of pulmonary hypertension (PH) in patients with chronic kidney disease (CKD) using meta-analysis.

**Methods:**

The PubMed, EmBase, and the Cochrane library were systematically searched for eligible studies from inception till May 2024. All of pooled analyses were performed using the random-effects model.

**Results:**

Fifty observational studies involving 17,558 CKD patients were selected. The prevalence of PH in CKD patients was 38% (95% confidence interval [CI]: 33%–43%), and the prevalence according to CKD status were 31% (95% CI: 20%–42%) for CKD (I-V), 39% (95% CI: 25%–54%) for end stage kidney disease (ESKD) (predialysis), 42% (95% CI: 35%–50%) for ESKD (hemodialysis), and 26% (95% CI: 19%–34%) for renal transplant. We noted the risk factors for PH in CKD included Black individuals (relative risk [RR]: 1.39; 95% CI: 1.18–1.63; *p* < 0.001), chronic obstructive pulmonary disease (RR: 1.48; 95% CI: 1.21-1.82; *p* < 0.001), cardiovascular disease history (RR: 1.62; 95% CI: 1.05–2.51; *p* = 0.030), longer dialysis (RR: 1.70; 95% CI: 1.18–2.46; *p* = 0.005), diastolic dysfunction (RR: 1.88; 95% CI: 1.38–2.55; *p* < 0.001), systolic dysfunction (RR: 3.75; 95% CI: 2.88–4.87; *p* < 0.001), and grade 5 CKD (RR: 5.64; 95% CI: 3.18–9.98; *p* < 0.001). Moreover, PH in CKD patients is also associated with poor prognosis, including all-cause mortality, major cardiovascular events, and cardiac death.

**Conclusion:**

This study systematically identified risk factors for PH in CKD patients, and PH were associated with poor prognosis. Therefore, patients with high prevalence of PH should be identified for treatment.

## Introduction

Pulmonary hypertension (PH) is a pathological condition characterized by persistently elevated pulmonary arterial pressure, primarily defined as a mean PAP ≥ 20 mmHg at rest and is caused by common left heart diseases or pulmonary disorders [1,2]. PH can be divided into primary and secondary, and it can progress to severe stages and with high risk of mortality if left untreated [3,4]. Up to 10% of older adults (> 65 years) are affected by PH [3]. Additionally, PH is common in patients with chronic kidney disease (CKD) primarily due to a high cardiac output state induced by arteriovenous fistulas, volume overload, and left ventricular stiffness [2,5]. Although PH in patients with CKD is associated with poor prognosis, its implications are still not given sufficient attention in clinical practice [6,7].

The prevalence of PH assessed by echocardiographic criteria ranges from 21–27% for patients with CKD, and up to 47% for patients with end stage kidney disease (ESKD) [8]. However, the mechanisms of PH in patients with CKD remain unclear, which may attribute to the imbalance of vasoconstrictors and vasodilators, left ventricular dysfunction, arteriovenous fistulas, mineral-and-bone disorders, anemia, and recurrent pulmonary embolisms in patients with CKD [9–12]. Moreover, PH in patients with CKD is associated with an elevated risk of all-cause and cardiovascular-related mortality [8,13]. Although several systematic reviews and meta-analyses have evaluated PH in patients at various stage of CKD [8,14,15], whether the predictors, prevalence and the prognostic role of PH in patients with CKD differ according to individuals’ characteristics are not well understood. Therefore, we aimed to assess the predictors, prevalence and prognostic role of PH in patients with CKD.

## Methodology

### Data sources, search strategy, and selection criteria

The Preferred Reporting Items for Systematic reviews and Meta-Analyses (PRISMA) guideline was applied to design and perform this systematic review and meta-analysis [16]. The study protocol is registered at the INPLASY register (INPLASY202320051). Observational studies reporting on the predictors, prevalence or prognostic role of PH for patients with CKD were potentially eligible for inclusion in this study, and the publication language and status were not restricted. We systematically searched PubMed, EmBase, and the Cochrane library to identify eligible studies throughout May 2024, and used ((chronic kidney disease OR CKD OR chronic renal failure OR chronic renal insufficiency OR CRF OR end stage kidney disease OR ESKD OR end stage renal disease OR ESRD OR dialysis) AND ((pulmonary AND (hypertension OR pressure)) as search terms. The detailed search strategy is provided in Supplementary file 1. We also reviewed the reference lists of relevant review or original articles to identify potential studies meeting the inclusion criteria.

The literature search and study selection were independently performed by 2 reviewers (QG, and LW), and inconsistent results between reviewers were resolved by an additional reviewer (CL) referring to the full text of the article. The inclusion criteria were: (1) All patients diagnosed with CKD, irrespective the disease status; (2) Study reported at least 1 of following outcomes: the prevalence of PH, predictors for PH, and the prognostic outcome of PH in patients with CKD, including all-cause mortality, major cardiovascular events (MACEs), and cardiac death; and (3) Study design: observational studies, including cross-sectional, retrospective cohort, and prospective cohort studies.

### Data extraction and quality assessment

The information abstracted from the included studies included: first authors’ surname, publication year, study design, country, sample size, mean age, male proportion, proportion of cardiovascular disease (CVD), diabetes mellitus (DM), and hypertension, ejection fraction (EF), CKD stage, definition of PH, PH diagnostic methods, predictors and prevalence of PH, associations of PH with the risk of all-cause mortality, major cardiovascular events (MACEs), or cardiac death in patients with CKD. Quality of the included studies was assessed using the Newcastle-Ottawa scale (NOS), which is a comprehensive and validated method for assessing the quality of observational studies in a meta-analysis [17]. The NOS includes 8 items in 3 subscales (selection: 4 items with 4 stars, comparability: 1 item with 2 stars, and outcome: 3 items with 3 stars), and the ‘star system’ for individual studies ranged from 0–9. The data collection and quality assessment were independently performed by two reviewers (CL and QG), and conflicts between reviewers were settled by group discussion until a consensus was reached.

### Statistical analysis

The prevalence of PH in patients with CKD was calculated based on PH events, sample size, and the pooled incidence and 95% confidence interval (CI) was calculated using the random-effects model. Moreover, the predictors for PH, and prognostic role of PH with the risk of all-cause mortality, MACEs, and cardiac death in patients with CKD were assigned a relative risk (RR) with 95% CI, and the pooled analyses were performed using the random-effects model, which considered the underlying variations across included studies [18,19]. *I^2^* and Cochren Q statistic were used to assess heterogeneity among included studies, and *I^2^* > 50.0% or *p* < 0.10 was consid­ered as significant heterogeneity [20,21]. Sensitivity analyses were performed for the predictors for PH and prognostic role of PH in patients with CKD to assess the robustness of pooled conclusion by sequentially removing single studies [22]. The prevalence of PH in patients with CKD was assessed according to CKD status, and subgroup analyses were performed based on study design and country. Moreover, subgroup analyses for the prognostic role of PH with the risk of all-cause mortality, MACEs, and cardiac death in patients with CKD were conducted based on study design, country, mean age, male, CVD, DM, hypertension, disease status, and PH definition, and the ratio of RR (RRR) with 95% CI calculated to compare the differences between subgroups, assuming the data met normal distribution [23]. Publication biases were assessed using the funnel plot, Egger, and Begg test results [24,25]. All reported *P* values for pooled effect estimates are two-sided, and *p* < 0.05 was considered statistically significant. Statistical analyses were performed using the software STATA (version 10.0; Stata Corporation, College Station, TX, USA).

## Results

### Search of the literature

**Figure 1.**
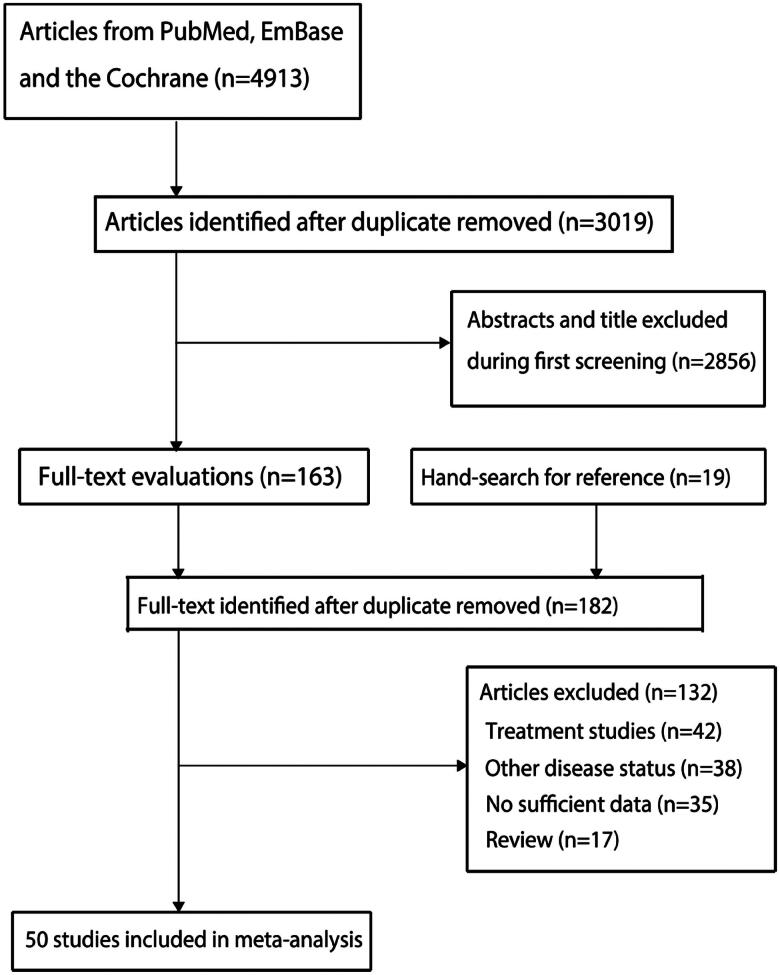
The PRISMA flowchart for literature search and study selection.

A total of 4,913 articles were identified from electronic searches, and 3,019 studies were retained after duplicate articles were removed. Further, 2,856 articles were excluded during initial title or abstract screening, while the remaining 163 articles were retrieved for full-text evaluations. Moreover, 19 additional articles were retrieved by manually reviewing the reference lists of original articles. After detailed evaluations, 132 articles were removed due to: treatment studies (*n* = 42), other disease status (*n* = 38), insufficient data (*n* = 35), reviews (*n* = 17). Ultimately, 50 studies were selected for the final meta-analysis [7,26–74]. The details of literature search and study selection process are illustrated in Figure 1.

### Study characteristics

The baseline characteristics of included studies and involved patients are presented in Table 1. A total of 17,558 CKD patients were identified from 50 studies, and the sample size ranged from 12 to 2,959. Of the included studies, 22 were prospective, while the remaining 28 were retrospective. Twelve studies included patients with CKD, 13 studies included patients with ESKD at predialysis, 28 studies included patients with ESKD at hemodialysis (HD), and five studies included patients with renal transplant. The methodological quality of individual studies are presented in Table 2; three studies had 9 stars, seven studies had 8 stars, 15 studies with 7 stars, 15 studies with 6 stars, and the remaining 10 studies with 5 stars.

**Table 1.** The baseline characteristics of identified studies and involved patients.

Study	Study design	Country	Sample size	Mean age (years)	Male (%)	CVD (%)	DM (%)	Hypertension (%)	EF (%)	CKD stage	Definition of PH	PH diagnostic methods	PH (%)
Yigla et al. (2003) [26]	Pro	Israel	58	58.8	53.4	NA	31.0	25.9	NA	ESKD (HD)	PASP ≥ 35 mmHg	ECHO	39.7
Kumbar et al. (2007) [27]	Retro	US	36	55.0	42.0	31.0	42.0	91.7	52.0	ESKD (HD)	PASP ≥ 35 mmHg	ECHO	42.0
Havlucu et al. (2007) [28]	Pro	Turkey	48	59.9	41.7	NA	25.0	37.5	59.1	ESKD (predialysis and HD)	PASP ≥ 35 mmHg	ECHO	47.9
Issa et al. (2008) [29]	Retro	US	215	55.0	61.0	39.0	46.0	NA	NA	Renal transplant	PASP ≥ 35 mmHg	ECHO	32.1
Abdelwhab et al. (2008) [30]	Retro	Egypt	76	50.8	56.6	NA	26.3	13.2	NA	ESKD (predialysis and HD)	PASP ≥ 35 mmHg	ECHO	39.5
Yigla et al. (2008) [31]	Pro	Israel	12	69.0	75.0	NA	50.0	25.0	NA	ESKD (predialysis)	PASP ≥ 35 mmHg	ECHO	42.0
Yigla et al. (2009) [32]	Retro	Israel	127	61.6	61.4	45.7	32.3	10.2	NA	ESKD (HD)	PASP ≥ 45 mmHg	ECHO	29.1
Ramasubbu et al. (2010) [33]	Pro	US	90	58.3	66.0	NA	NA	NA	NA	ESKD (HD)	TRV ≥ 2.5 m/d	ECHO	46.7
Kiykim et al. (2010) [34]	Pro	Turkey	74	44.6	56.8	NA	NA	NA	64.0	ESKD (HD)	PASP ≥ 30 mmHg	ECHO	68.8
Zlotnick et al. (2010) [35]	Retro	US	55	53.3	62.0	20.0	36.0	87.0	61.7	Renal transplant	PASP ≥ 35 mmHg	ECHO	38.2
Agarwal et al. (2012) [36]	Pro	US	288	54.4	63.9	34.0	45.1	80.9	NA	ESKD (HD)	PASP ≥ 35 mmHg	ECHO	38.2
Yoo et al. (2012) [37]	Retro	Brazil	75	56.2	60.0	NA	NA	NA	NA	ESKD (HD)	PASP ≥ 35 mmHg	ECHO	30.6
Pabst et al. (2012) [38]	Pro	Germany	62	69.5	58.1	19.4	39.0	58.1	57.5	ESKD (predialysis and HD)	PASP ≥ 30 mmHg	ECHO	74.0
Said et al. (2012) [39]	Pro	Egypt	41	48.0	60.0	NA	17.0	83.0	65.0	ESKD (predialysis)	PASP ≥ 35 mmHg	ECHO	36.5
Stallworthy et al. (2013) [40]	Retro	New Zealand	739	53.0	64.0	NA	32.0	NA	NA	Renal transplant	PASP ≥ 30 mmHg	ECHO	18.0
El-Azeem et al. (2013) [41]	Retro	Egypt	99	45.7	54.5	NA	40.4	31.3	52.4	ESKD (predialysis and HD)	PASP ≥ 35 mmHg	ECHO	34.3
Green et al. (2014) [42]	Pro	UK	323	61.5	62.8	20.1	33.7	NA	68.0	ESKD (HD)	PASP ≥ 35 mmHg	ECHO	17.0
Li et al. (2014) [43]	Pro	China	278	58.0	53.6	30.6	33.8	91.1	63.3	ESKD (HD)	PASP ≥ 35 mmHg	ECHO	35.2
Yang et al. (2014) [44]	Retro	China	128	53.4	48.4	NA	NA	NA	NA	CKD (I–III)	PASP ≥ 35 mmHg	ECHO	28.9
Li et al. (2014) [45]	Retro	China	2,351	52.5	54.8	27.4	27.7	76.6	64.8	CKD (I–V)	PASP ≥ 35 mmHg	ECHO	18.1
Bolignano et al. (2015) [46]	Pro	Italy, Germany	468	64.0	60.0	30.0	35.0	NA	63.0	CKD (II–IV)	PASP ≥ 35 mmHg	ECHO	23.0
Kim et al. (2015) [47]	Retro	Korea	172	56.3	50.0	8.7	43.0	88.4	NA	ESKD (HD)	PASP ≥ 37 mmHg	ECHO	36.6
Xu et al. (2015) [48]	Retro	China	618	50.5	57.0	33.5	28.6	NA	65.0	ESKD (HD)	PASP ≥ 35 mmHg	ECHO	16.0
Babua et al. (2015) [49]	Retro	Uganda	217	42.8	51.1	NA	NA	NA	NA	CKD (I–V)	PASP ≥ 35 mmHg	ECHO	22.1
Genctoy et al. (2015) [50]	Retro	Turkey	190	61.1	61.1	NA	29.5	NA	58.6	CKD (I–IV)	PASP ≥ 35 mmHg	ECHO	35.9
Hsieh et al. (2016) [51]	Pro	China	160	66.4	41.9	48.8	48.8	48.1	61.8	ESKD (HD)	PASP ≥ 35 mmHg	ECHO	31.9
Navaneethan et al. (2016) [52]	Pro	US	2,959	59.6	53.9	36.9	48.3	NA	53.0	CKD (I–V)	PASP ≥ 35 mmHg	ECHO	21.1
Reque et al. (2016) [53]	Pro	Spain	211	69.0	55.6	42.7	31.5	86.6	NA	ESKD (HD)	PASP ≥ 35 mmHg	ECHO	43.9
Reque et al. (2017) [54]	Pro	Spain	353	67.0	54.7	26.9	43.6	65.1	NA	CKD (III–V)	PASP ≥ 35 mmHg	ECHO	26.6
Suresh et al. (2017) [55]	Retro	India	80	43.5	62.5	NA	38.8	75.0	NA	CKD (III–V)	PASP ≥ 35 mmHg	ECHO	43.8
Selvaraj et al. (2017) [56]	Pro	US	408	63.0	30.0	18.0	39.0	78.0	61.0	CKD (I–V)	PASP ≥ 35 mmHg	ECHO	21.6
O’Leary et al. (2017) [57]	Retro	US	1,873	65.6	48.5	NA	22.2	78.3	NA	CKD (III–V)	mPAP ≥ 25 mmHg	RHC	67.6
Zhang et al. (2018) [58]	Retro	China	705	48.1	55.3	NA	NA	NA	62.1	CKD (I–V)	PASP ≥ 35 mmHg	ECHO	47.4
Miri et al. (2018) [59]	Retro	Iran	50	33.9	58.8	NA	10.0	8.0	54.6	ESKD (HD)	PASP ≥ 35 mmHg	ECHO	22.0
Singh et al. (2018) [60]	Pro	India	50	49.6	50.0	14.0	46.0	86.0	55.0	ESKD (HD)	RVSP ≥ 25 mmHg	ECHO	34.0
Tudoran et al. (2020) [61]	Pro	Romania	51	57.6	52.9	NA	NA	NA	52.1	ESKD (HD)	PASP ≥ 35 mmHg	ECHO	52.9
Orihuela et al. (2020) [62]	Pro	Mexico	177	53.6	59.3	NA	70.6	24.9	NA	ESKD (predialysis)	PASP ≥ 35 mmHg	ECHO	38.9
Nithiya et al. (2020) [63]	Pro	India	113	50.1	63.7	6.2	39.8	53.1	NA	ESKD (predialysis and HD)	PASP ≥ 37 mmHg	ECHO	48.7
Obi et al. (2020) [64]	Retro	US	733	50.1	NA	NA	NA	NA	62.0	Renal transplant	PASP ≥ 35 mmHg	ECHO	15.6
Zhang et al. (2020) [65]	Retro	China	1,092	52.0	59.8	6.5	42.1	9.2	67.6	CKD (I–V)	PASP ≥ 35 mmHg	ECHO	15.9
Engole et al. (2020) [66]	Retro	Congo	85	52.6	67.1	NA	37.6	91.8	64.4	ESKD (HD)	PASP ≥ 35 mmHg	ECHO	29.4
Li et al. (2020) [67]	Retro	China	141	40.4	66.7	NA	NA	77.3	65.2	ESKD (predialysis and HD)	PASP ≥ 35 mmHg	ECHO	16.3
Rroji et al. (2021) [7]	Pro	Albania	125	52.4	60.0	15.2	19.2	NA	57.0	ESKD (HD)	PASP ≥ 35 mmHg	ECHO	28.0
Mann et al. (2021) [68]	Retro	India	100	32.0	NA	NA	21.0	NA	42.4	ESKD (predialysis and HD)	mPAP ≥ 25 mmHg	RHC	61.0
Alici et al. (2022) [69]	Retro	Somalia	143	54.2	45.5	NA	50.3	56.6	51.9	ESKD (HD)	PASP ≥ 35 mmHg	ECHO	51.0
Anandan et al. (2022) [70]	Retro	India	100	50.4	62.0	NA	64.0	92.0	63.5	ESKD (predialysis and HD)	mPAP ≥ 30 mmHg	ECHO	89.0
Rabih et al. (2022) [71]	Retro	USA	350	51.0	60.0	NA	28.0	76.0	60.0	Renal transplant	PASP ≥ 35 mmHg	ECHO	33.0
Singh et al. (2022) [72]	Retro	India	378	51.6	65.6	NA	37.6	23.0	NA	ESKD (predialysis)	PASP ≥ 35 mmHg	ECHO	12.2
Liu et al. (2023) [73]	Pro	China	181	57.9	52.5	64.1	38.7	78.5	57.8	ESKD (HD)	PASP ≥ 35 mmHg	ECHO	30.9
Gaur et al. (2023) [74]	Retro	India	100	54.5	54.0	NA	35.0	32.0	NA	ESKD (predialysis and HD)	PASP ≥ 35 mmHg	ECHO	47.0

* CKD: chronic kidney disease; CVD: cardiovascular disease; DM: diabetes mellitus; ECHO: Echocardiography; EF: ejection fraction; ESKD: end-stage kidney disease; HD: hemodialysis; NA: not available; mPAP: mean pulmonary artery pressure; PAH: pulmonary arterial hypertension; PASP: pulmonary artery systolic pressure; Pro: prospective; Retro: retrospective; RHC: right heart catheterization.

**Table 2.** Quality scores of prospective cohort studies using Newcastle-Ottawa scale.

Study	Selection				Comparability	Outcome			NOS
	Representativeness of the exposed cohort	Selection of the non exposed cohort	Ascertainment of exposure	Demonstration that outcomes was not present at start of study	Comparability on the basis of the design or analysis	Assessment of outcome	Adequate follow-up duration	Adequate follow-up rate	Overall score
Yigla et al. (2003) [26]	0	1	1	1	1	1	0	0	5
Kumbar et al. (2007) [27]	0	1	1	1	1	1	0	0	5
Havlucu et al. (2007) [28]	0	1	1	1	1	1	0	0	5
Issa et al. (2008) [29]	1	1	1	1	1	1	1	0	7
Abdelwhab et al. (2008) [30]	0	1	1	1	1	1	1	0	6
Yigla et al. (2008) [31]	0	1	1	1	1	1	0	0	5
Yigla et al. (2009) [32]	1	1	1	1	1	1	0	1	7
Ramasubbu et al. (2010) [33]	0	1	1	1	1	1	0	0	5
Kiykim et al. (2010) [34]	0	1	1	1	1	1	1	0	6
Zlotnick et al. (2010) [35]	0	1	1	1	1	1	0	0	5
Agarwal et al. (2012) [36]	1	1	1	1	1	1	1	1	8
Yoo et al. (2012) [37]	1	1	1	1	1	1	0	1	7
Pabst et al. (2012) [38]	0	1	1	1	1	1	1	0	6
Said et al. (2012) [39]	0	1	1	1	1	1	1	0	6
Stallworthy et al. (2013) [40]	1	1	1	1	1	1	1	1	8
El-Azeem et al. (2013) [41]	0	1	1	1	1	1	0	1	6
Green et al. (2014) [42]	1	1	1	1	1	1	1	1	8
Li et al. (2014) [43]	1	1	1	1	1	1	1	1	8
Yang et al. (2014) [44]	1	1	1	1	1	1	0	1	7
Li et al. (2014) [45]	1	1	1	1	1	1	0	0	6
Bolignano et al. (2015) [46]	1	1	1	1	1	1	0	1	7
Kim et al. (2015) [47]	1	1	1	1	1	1	0	1	7
Xu et al. (2015) [48]	1	1	1	1	1	1	1	1	8
Babua et al. (2015) [49]	1	1	1	1	1	1	0	0	6
Genctoy et al. (2015) [50]	1	1	1	1	1	1	0	0	6
Hsieh et al. (2016) [51]	1	1	1	1	1	1	0	1	7
Navaneethan et al. (2016) [52]	1	1	1	1	2	1	1	1	9
Reque (2016) et al. [53]	1	1	1	1	1	1	1	1	8
Reque et al. (2017) [54]	1	1	1	1	1	1	0	1	7
Suresh et al. (2017) [55]	0	1	1	1	1	1	0	0	5
Selvaraj et al. (2017) [56]	1	1	1	1	1	1	0	1	7
O’Leary et al. (2017) [57]	1	1	1	1	1	1	0	0	6
Zhang et al. (2018) [58]	1	1	1	1	2	1	1	1	9
Miri et al. (2018) [59]	0	1	1	1	1	1	0	0	5
Singh et al. (2018) [60]	0	1	1	1	1	1	0	0	5
Tudoran et al. (2020) [61]	0	1	1	1	1	1	0	0	5
Orihuela et al. (2020) [62]	1	1	1	1	1	1	0	1	7
Nithiya et al. (2020) [63]	1	1	1	1	1	1	0	1	7
Obi et al. (2020) [64]	1	1	1	1	1	1	0	1	7
Zhang et al. (2020) [65]	1	1	1	1	1	1	0	1	7
Engole et al. (2020) [66]	0	1	1	1	1	1	1	0	6
Li et al. (2020) [67]	1	1	1	1	1	1	0	0	6
Rroji et al. (2021) [7]	1	1	1	1	2	1	1	1	9
Mann et al. (2021) [68]	1	1	1	1	1	1	0	0	6
Alici et al. (2022) [69]	1	1	1	1	1	1	0	1	7
Anandan et al. (2022) [70]	1	1	1	1	1	1	0	0	6
Rabih et al. (2022) [71]	1	1	1	1	1	1	0	1	7
Singh et al. (2022) [72]	1	1	1	1	1	1	0	0	6
Liu et al. (2023) [73]	1	1	1	1	1	1	1	1	8
Gaur et al. (2023) [74]	1	1	1	1	1	1	0	0	6

### Prevalence of PH

**Figure 2.**
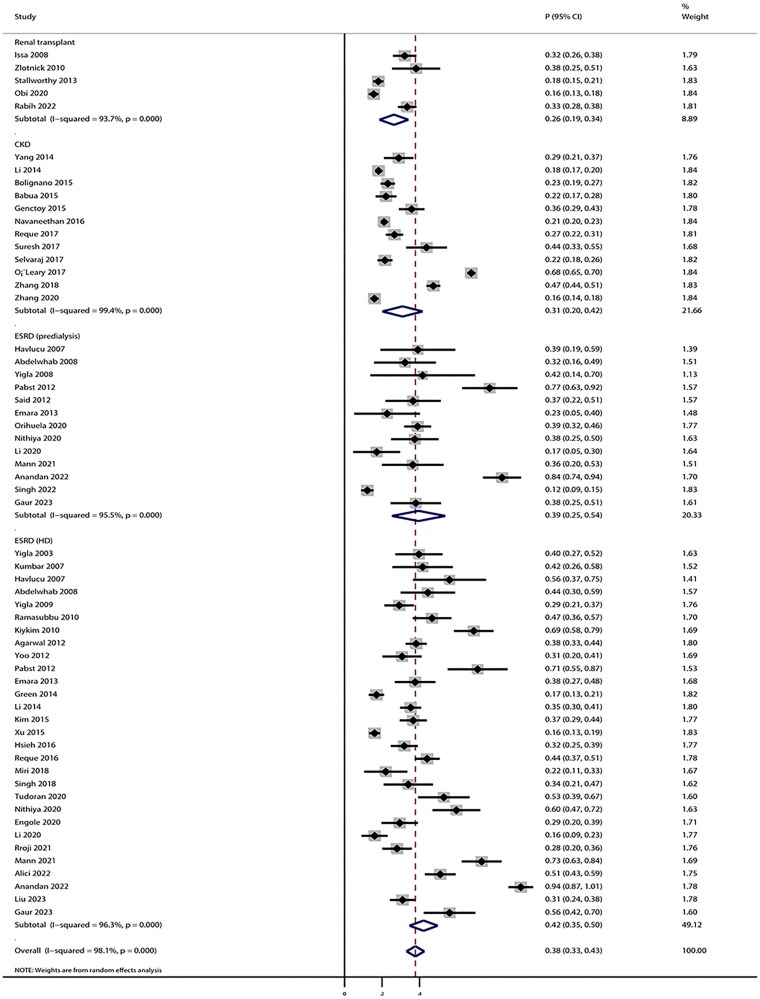
The pooled prevalence of pulmonary hypertension in patients with any stage of chronic kidney disease. The X-axis represents the incidence rate, with 0 as the baseline, ‘overall’ denotes the pooled prevalence of PH across all studies, and ‘subtotal’ signifies the prevalence of PH among patients with specific CKD stage. P: proportion; CI: confidence interval.

After pooling all included studies, we noted that prevalence of PH in patients with CKD was 38% (95% CI: 33%–43%), and the prevalence according to CKD status were 31% (95% CI: 20%–42%) for CKD (I-V), 39% (95% CI: 25%–54%) for ESKD (predialysis), 42% (95% CI: 35%–50%) for ESKD (HD), and 26% (95% CI: 19%–34%) for renal transplant (Figure 2). There was significant heterogeneity across included studies for overall analysis (*I^2^* = 98.1%; *p* < 0.001), CKD (I–V) (*I^2^* = 99.4%; *p* < 0.001), ESKD (predialysis) (*I^2^* = 95.5%; *p* < 0.001), ESKD (HD) (*I^2^* = 96.3%; *p* < 0.001), and renal transplant patients (*I^2^* = 93.7%; *p* < 0.001). Subgroup analyses for the prevalence of PH are presented in Table 3. We noted that the prevalence of PH in patients with CKD (I-V) was higher in pooled retrospective studies, and studies performed in Europe and Asia (Turkey), and North America. Moreover, the prevalence of PH in patients with ESKD (predialysis) was higher in prospective studies and studies performed in Europe. Similarly, the prevalence of PH in patients with ESKD (HD) was higher in pooled prospective studies and studies performed in Europe and Asia (Turkey). The prevalence of PH in patients with renal transplant was higher only in the studies performed in North America.

**Table 3.** Subgroup analyses for the prevalence of PH.

CKD status	Factors	Subgroups	No. studies	Prevalence and 95%CI	Heterogeneity (*I^2^*)	Q statistic	*P* value between subgroups
CKD	Study design	Prospective	4	0.22 (0.20–0.25)	46.0	0.136	<0.001
		Retrospective	8	0.35 (0.18–0.52)	99.6	<0.001	
	Country	Africa	1	0.22 (0.17–0.28)	–	–	<0.001
		Asia	5	0.30 (0.19–0.42)	98.4	<0.001	
		Europe	2	0.25 (0.21–0.28)	26.1	0.245	
		Europe and Asia	1	0.36 (0.29–0.43)	–	–	
		North America	3	0.37 (0.03–0.70)	99.8	<0.001	
ESKD (predialysis)	Study design	Prospective	6	0.45 (0.33–0.58)	79.0	<0.001	<0.001
		Retrospective	7	0.35 (0.12–0.57)	96.8	<0.001	
	Country	Asia	7	0.38 (0.15–0.61)	96.9	<0.001	<0.001
		Asia and Africa	3	0.31 (0.22–0.41)	0.0	0.490	
		Europe	1	0.77 (0.63–0.92)	–	–	
		Europe and Asia	1	0.39 (0.19–0.59)	–	–	
		North America	1	0.39 (0.32–0.46)	–	–	
ESKD (HD)	Study design	Prospective	15	0.43 (0.35–0.50)	92.1	<0.001	0.103
		Retrospective	14	0.41 (0.27–0.55)	97.8	<0.004	
	Country	Africa	2	0.40 (0.19–0.62)	91.1	0.001	<0.001
		Asia	14	0.41 (0.28–0.54)	97.7	<0.001	
		Asia and Africa	2	0.40 (0.31–0.49)	0.0	0.463	
		Europe	5	0.42 (0.25–0.58)	95.6	<0.001	
		Europe and Asia	2	0.65 (0.54–0.77)	23.6	0.253	
		North America	3	0.40 (0.36–0.45)	1.7	0.362	
		South America	1	0.31 (0.20–0.41)	–	–	
Renal transplant	Study design	Prospective	0	–	–	–	–
		Retrospective	5	0.26 (0.19–0.34)	93.7	<0.001	
	Country	North America	4	0.29 (0.17–0.41)	95.0	<0.001	0.051
		Oceania	1	0.18 (0.15–0.21)	–	–	

### Predictors for PH

**Figure 3.**
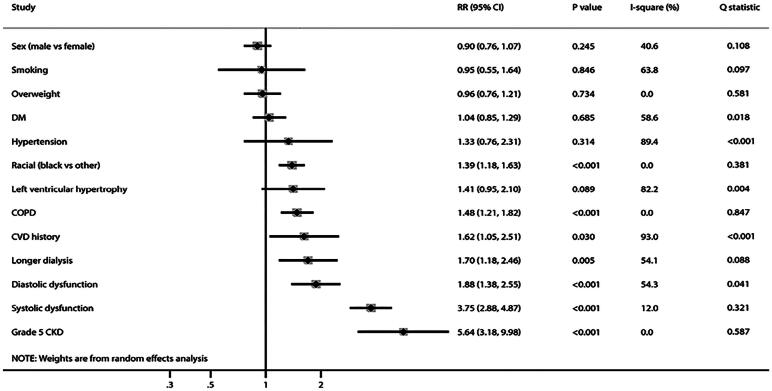
The predictors for pulmonary hypertension in patients with chronic kidney disease. The X-axis represents the impact of specific factors on the risk of PH occurrence, where an RR less than 1 indicates a protective factor, and an RR greater than 1 signifies a risk factor. RR: relative risk; CI: confidence interval.

The predictors for PH in patients with CKD are summarized in Figure 3. We noted that Black individuals (RR: 1.39; 95% CI: 1.18–1.63; *p* < 0.001), chronic obstructive pulmonary disease (RR: 1.48; 95% CI: 1.21–1.82; *p* < 0.001), cardiovascular disease history (RR: 1.62; 95% CI: 1.05–2.51; *p* = 0.030), longer dialysis (RR: 1.70; 95% CI: 1.18–2.46; *p* = 0.005), diastolic dysfunction (RR: 1.88; 95% CI: 1.38–2.55; *p* < 0.001), systolic dysfunction (RR: 3.75; 95% CI: 2.88–4.87; *p* < 0.001), and grade 5 CKD (RR: 5.64; 95% CI: 3.18–9.98; *p* < 0.001) were associated to have an elevated risk of PH in patients with CKD. However, sex, smoking, overweight, diabetes mellitus, hypertension, and left ventricular hypertrophy did not affect the risk of PH in patients with CKD.

### All-cause mortality

**Figure 4.**
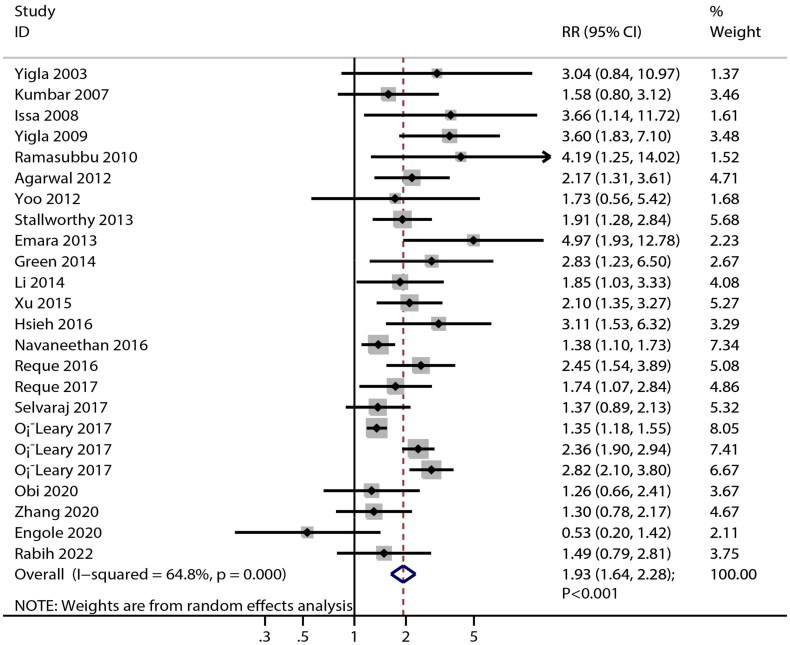
Association of pulmonary hypertension in patients with chronic kidney disease with the risk of all-cause mortality. The X-axis represents the association of PH with the risk of all-cause mortality, where an RR less than 1 indicates a protective factor, and an RR greater than 1 signifies a risk factor. RR: relative risk; CI: confidence interval.

In total, 22 studies reported a significant association of PH with the risk of all-cause mortality in patients with CKD in all subgroups (Figure 4 and Table 4). The strength of this association in patients with history of CVD was higher than in those without history of CVD (RRR: 1.35; 95% CI: 0.95-1.94; *p* = 0.098). Patients with CKD with PH were associated with an increased risk of all-cause mortality (RR: 1.93; 95% CI: 1.64-2.28; *p* < 0.001), with significant heterogeneity observed across included studies (*I^2^*=64.8%; *p* < 0.001). Sensitivity analysis indicated the pooled conclusion was robust and was not altered by sequential removing of single studies (Figure S1).

**Table 4.** Subgroup analyses for all-cause mortality, MACEs, and cardiac death.

Outcomes	Factors	Subgroups	No. studies	RR and 95%CI	*P* value	*I^2^*(%)	Q statistic	*P* value between subgroups	*RRR between subgroups*
All-cause mortality	Study design	Prospective	10	1.92 (1.54–2.40)	<0.001	38.7	0.100	0.924	1.02 (0.73-1.41)
		Retrospective	14	1.89 (1.49–2.40)	<0.001	74.4	<0.001		
	Country	Eastern	6	2.16 (1.58–2.96)	<0.001	33.2	0.187	0.424	1.16 (0.80-1.68)
		Western	18	1.86 (1.54–2.25)	<0.001	69.3	<0.001		
	Mean age (years)	≥60.0	9	2.17 (1.64–2.86)	<0.001	81.1	<0.001	0.206	1.25 (0.89-1.76)
		<60.0	15	1.74 (1.43–2.13)	<0.001	37.2	0.073		
	Male (%)	≥60.0	9	2.08 (1.50–2.89)	<0.001	43.1	0.080	0.683	1.08 (0.74-1.59)
		<60.0	14	1.92 (1.57–2.34)	<0.001	72.9	<0.001		
	CVD (%)	≥30.0	9	2.10 (1.64–2.69)	<0.001	49.4	0.045	0.098	1.35 (0.95-1.94)
		<30.0	4	1.55 (1.20–2.02)	0.001	0.0	0.394		
	DM (%)	≥30.0	16	1.93 (1.57–2.36)	<0.001	50.9	0.010	0.921	0.98 (0.65-1.47)
		<30.0	5	1.97 (1.39–2.80)	<0.001	87.4	<0.001		
	Hypertension (%)	≥50.0	11	1.79 (1.41–2.26)	<0.001	75.5	<0.001	0.150	0.65 (0.36-1.17)
		<50.0	5	2.75 (1.61–4.70)	<0.001	59.2	0.044		
	Disease status	CKD	7	1.70 (1.32–2.19)	<0.001	82.9	<0.001	0.091; 0.906; 0.149	0.74 (0.53-1.05); 0.98 (0.67-1.44); 1.32 (0.91-1.91)
		ESKD	13	2.29 (1.81–2.90)	<0.001	29.9	0.145		
		Renal transplant	4	1.74 (1.30–2.32)	<0.001	0.0	0..98		
	PH definition	PASP≥ 35 mmHg	18	1.79 (1.50–2.14)	<0.001	38.4	0.050	0.243	0.79 (0.53-1.05)
		Other	6	2.27 (1.59–3.25)	<0.001	86.7	<0.001		
MACE	Study design	Prospective	7	1.95 (1.45–2.61)	<0.001	64.6	0.010	0.139	0.56 (0.26-1.21)
		Retrospective	2	3.51 (1.71–7.22)	0.001	0.0	0.759		
	Country	Eastern	4	2.62 (1.94–3.55)	<0.001	0.0	0.770	0.074	1.50 (0.96-2.33)
		Western	5	1.75 (1.26–2.41)	0.001	57.3	0.052		
	Mean age (years)	≥60.0	5	2.20 (1.72–2.82)	<0.001	0.0	0.687	0.880	1.05 (0.57-1.92)
		<60.0	4	2.10 (1.21–3.66)	0.009	75.0	0.007		
	Male (%)	≥60.0	1	1.75 (1.05–2.91)	0.031	–	–	0.487	0.81 (0.44-1.48)
		<60.0	8	2.17 (1.56–3.01)	<0.001	66.9	0.004		
	CVD (%)	≥30.0	5	1.82 (1.30–2.56)	0.001	68.7	0.012	0.122	0.67 (0.41-1.11)
		<30.0	4	2.70 (1.87–3.90)	<0.001	0.0	0.850		
	DM (%)	≥30.0	8	2.00 (1.50–2.66)	<0.001	61.8	0.011	0.201	0.51 (0.18-1.43)
		<30.0	1	3.90 (1.46–10.42)	0.007	–	–		
	Hypertension (%)	≥50.0	6	2.36 (1.83–3.05)	<0.001	0.0	0.816	0.624	0.87 (0.50-1.51)
		<50.0	1	2.71 (1.66–4.43)	<0.001	–	–		
	Disease status	CKD	5	1.96 (1.30–2.95)	0.001	68.6	0.013	0.495	0.84 (0.51-1.38)
		ESKD	4	2.33 (1.76–3.08)	<0.001	0.0	0.690		
	PH definition	PASP≥ 35 mmHg	8	2.04 (1.52–2.74)	<0.001	64.8	0.006	0.452	0.66 (0.22-1.97)
		Other	1	3.11 (1.08–8.96)	0.036	–	–		
Cardiac death	Study design	Prospective	2	2.76 (1.38–5.52)	0.004	0.0	0.461	0.445	1.35 (0.62-2.94)
		Retrospective	3	2.04 (1.44–2.88)	< 0.001	0.0	0.407		
	Country	Eastern	3	2.34 (1.62–3.38)	< 0.001	0.0	0.864	0.843	1.11 (0.38-3.24)
		Western	2	2.10 (0.77–5.72)	0.146	50.5	0.155		
	Mean age (years)	≥60.0	1	4.24 (1.12–16.07)	0.034	–	–	0.308	2.04 (0.52-8.02)
		<60.0	4	2.08 (1.51–2.86)	< 0.001	0.0	0.592		
	Male (%)	≥60.0	2	2.10 (0.77–5.72)	0.146	50.5	0.155	0.843	0.90 (0.31-2.61)
		<60.0	3	2.34 (1.62–3.38)	< 0.001	0.0	0.864		
	CVD (%)	≥30.0	2	2.52 (1.59–4.00)	< 0.001	0.0	0.847	0.403	1.31 (0.69-2.48)
		<30.0	3	1.92 (1.24–2.99)	0.004	7.0	0.341		
	DM (%)	≥30.0	4	2.00 (1.38–2.90)	< 0.001	0.0	0.502	0.446	0.77 (0.39-1.51)
		<30.0	1	2.60 (1.48–4.56)	0.001	–	–		
	Hypertension (%)	≥50.0	1	2.36 (1.05–5.31)	0.038	–	–	0.799	1.14 (0.41-3.13)
		<50.0	1	2.07 (1.13–3.79)	0.018	–	–		
	Disease status	CKD	1	2.07 (1.13–3.79)	0.018	–	–	0.510; 0.430; 0.127	0.78 (0.37-1.64); 1.43 (0.59-3.46); 1.83 (0.84-4.00)
		ESKD	3	2.66 (1.72–4.12)	< 0.001	0.0	0.756		
		Renal transplant	1	1.45 (0.76–2.76)	0.257	–	–		
	PH definition	PASP≥ 35 mmHg	4	2.44 (1.72–3.48)	< 0.001	0.0	0.801	0.165	1.68 (0.81-3.51)
		Other	1	1.45 (0.76–2.76)	0.257	–	–		

### Major cardiovascular events

**Figure 5.**
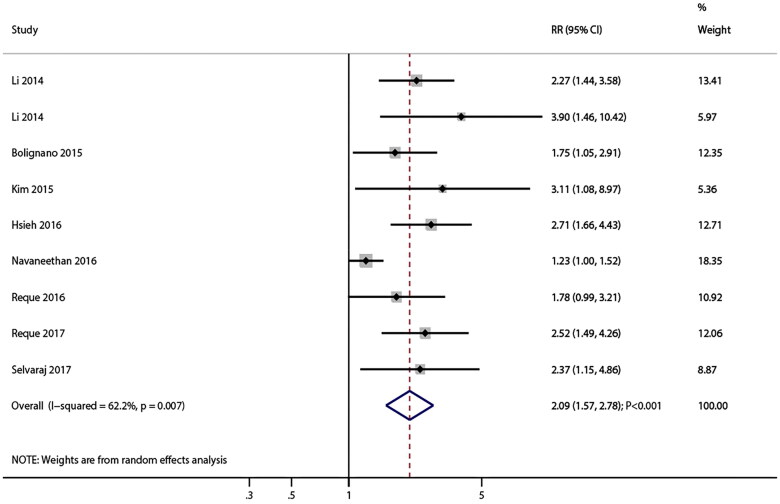
Association of pulmonary hypertension in patients with chronic kidney disease with the risk of major cardiovascular events. The X-axis represents the association of PH with the risk of major cardiovascular events, where an RR less than 1 indicates a protective factor, and an RR greater than 1 signifies a risk factor. RR: relative risk; CI: confidence interval.

A total of nine studies reported an association of PH with the risk of MACEs in patients with CKD (Figure 5) which was statistically significant in all subgroups. The strength of this association in the subgroup of Eastern countries was higher than that in Western countries (RRR: 1.50; 95% CI: 0.96–2.33; *p* = 0.074) (Table 4). We noted that patients with CKD with PH were associated with an increased risk of MACEs (RR: 2.09; 95% CI: 1.57–2.78; *p* < 0.001), with significant heterogeneity was observed across included studies (*I^2^*=62.2%; *p* = 0.007). The pooled conclusion was stable even when after sensitivity analysis by removing any particular study (Figure S2).

### Cardiac death

**Figure 6.**
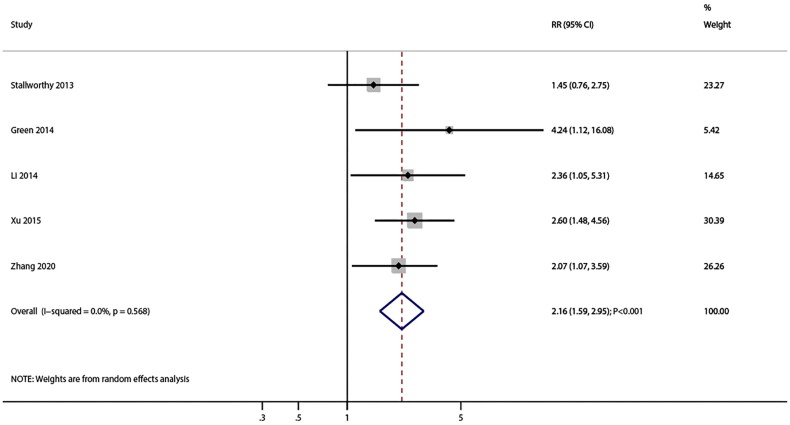
Association of pulmonary hypertension in patients with chronic kidney disease with the risk of cardiac death. The X-axis represents the association of PH with the risk of cardiac death, where an RR less than 1 indicates a protective factor, and an RR greater than 1 signifies a risk factor. RR: relative risk; CI: confidence interval.

In total, five studies reported the association of PH with the increased risk of cardiac death in patients with CKD (RR: 2.16; 95%CI: 1.59–2.95; *p* < 0.001) and no evidence of heterogeneity among included studies (*I^2^*=0.0%; *p* = 0.568) (Figure 6). Sensitivity analysis indicated the pooled conclusion for cardiac death was stable and did not change after excluding any specific study (Figure S3). Although significant association between PH and cardiac death was observed in most subgroups, PH was not associated with the risk of cardiac death in pooled studies performed in Western countries, percentage of male ≥ 60.0%, studies including renal transplant patients, and when other PH definition criteria were used (Table 4).

### Publication bias

**Figure 7.**
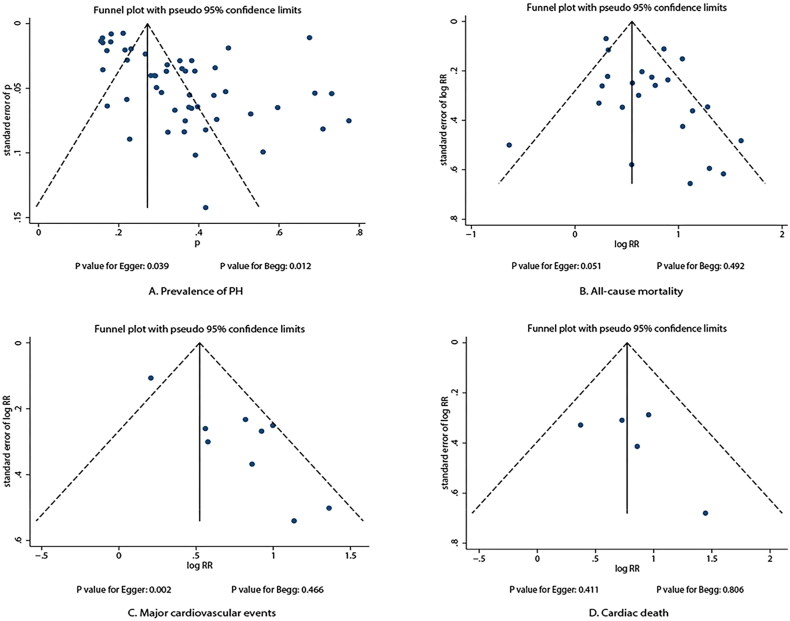
Funnel plots for the prevalence and prognostic role of pulmonary hypertension in patients with chronic kidney disease.

Publication bias for the prevalence of PH and prognostic role of PH with the risk of all-cause mortality, MACEs, and cardiac death are illustrated in Figure 7. We noted potential significant publication bias for the prevalence of PH in patients with CKD (*P* value for Egger test: 0.039; *P* value for Begg test: 0.012). Moreover, although the Begg test indicated no significant publication bias for MACEs (*p* = 0.466), the Egger test did indicate potential significant publication bias (*p* = 0.002). Furthermore, there were no significant publication biases for all-cause mortality (*P* value for Egger test: 0.051; *P* value for Begg test: 0.492) and cardiac death (*P* value for Egger test: 0.411; *P* value for Begg test: 0.806)

## Discussion

### Summary of results

Our results show that the prevalence of PH differs in patients at various CKD stages, and the prognostic role of PH in CKD patients may affected by individuals’ characteristics, which should be explored to identify high risk patients. We identified 50 studies and involving 17,558 patients with CKD across a broad range of patient characteristics. The results show that risk of PH in patients with CKD is affected by ethnicity (particularly in Black individuals), chronic obstructive pulmonary disease, cardiovascular disease history, longer dialysis, diastolic dysfunction, systolic dysfunction, and grade 5 CKD. Patients with CKD presenting with PH were associated with an increased risk of all-cause mortality, MACEs, and cardiac death. Additionally, the role of PH with all-cause mortality may be affected by history of CVD, while country may affect the strength of PH association with the risk of MACEs.

### Prevalence of PH in CKD

Our study found that overall PH prevalence in any stage CKD was 38%, which was slightly higher than previous studies [14]. Moreover, we noted the overall PH prevalence in patients with CKD (I-V), ESKD (predialysis), ESKD (HD), and renal transplant was 31%, 39%, 42%, and 26%, respectively. The prevalence of PH in patients with CKD (I-V) without dialysis was 31%, similar to a previous study [15]. Additionally, the progression of PH in patients with CKD can be attributed to: (1) left ventricular hypertrophy and diastolic dysfunction which are more common in patients with CKD [75]; (2) increase in the systemic and pulmonary vascular resistance caused by endothelial dysfunction in patients with CKD [76]; (3) vascular calcification in patients with CKD which is significantly associated with pulmonary vascular remodeling [77]; and (4) volume overload, sleep-disordered breathing, stiffening, and severe anemia in patients with CKD which induce PH progression [78]. Furthermore, PH leads to an increased pressure load on the right side of the heart, causing right ventricular hypertrophy and dysfunction, which may in turn affect the function of the left ventricle, reducing cardiac output. The diminished pumping capacity of the heart decreases renal perfusion, impacting glomerular filtration rate, and thereby exacerbating the progression of CKD [79,80]. Pulmonary circulation disturbances caused by PH can reduce the efficiency of oxygen exchange in the lungs, leading to systemic hypoxemia. Hypoxia not only directly injures renal tissue but also stimulates vasoconstriction, promotes inflammatory responses, and accelerates the fibrotic process, thereby further hastening the progression of CKD [74]. Stratified analyses found that PH prevalence in patients with CKD (I-V) was high when pooled studies were retrospective, and the population included patients from Europe and Asia (Turkey), or North America. PH occurrence in patients with ESKD (predialysis) was high in pooled prospective studies, and studies performed in Europe. Similarly, PH prevalence in patients with ESKD (HD) was higher in prospective studies, and studies performed in Europe and Asia (Turkey). Whereas PH occurrence in patients after renal transplant was higher in studies performed in North America. The primary reasons for the discrepancies in reporting the incidence of PH between retrospective and prospective studies are attributed to recall bias, the representativeness of the samples, and the accuracy of exposure measurement. Moreover, variations in genetic backgrounds, lifestyles, dietary habits, levels of environmental pollution, and accessibility to medical resources among CKD patients across different countries can influence the incidence and progression of PH. These results provide comprehensive epidemiological data to determine patients at high risk for PH, who should be cautiously monitored to improve the prognosis of CKD.

### Predictors for PH in CKD

Our study found the risk factors for PH in patients with CKD included Black individuals, chronic obstructive pulmonary disease, cardiovascular disease history, longer dialysis, diastolic dysfunction, systolic dysfunction, and grade 5 CKD. Possible explanations for these results are: (1) Black individuals exhibit higher levels of endothelin-1 during stress responses, which can potentially damage vascular endothelial function. Endothelial cells, which regulate the balance between vasoconstriction and vasodilation, encounter enhanced smooth muscle contraction and a decrease in the bioavailability of nitric oxide as a result of excessive endothelin-1 secretion. This imbalance reduces the ability of vessels to dilate, thereby contributing to increased PH [81,82]; (2) PH was a frequent complication of chronic obstructive pulmonary disease with insidious onset and nonspecific symptoms [83]; (3) the relationship between PH and cardiovascular disease have demonstrated [84]; and (4) CKD severity is significantly related to the risk of PH, which was also observed for the prevalence of PH in CKD patients.

### Prognostic role of PH in CKD

In summary, the results indicated PH in patients with CKD is associated with an increased risk of all-cause mortality, MACEs, and cardiac death, consistent with prior meta-analyses [8,14]. Tang et al. identified 16 studies and found the overall PH prevalence in CKD and ESKD patients was 23% which was associated with a substantially increased all-cause mortality, MACEs, and cardiac death. Moreover, they showed that the risk of all-cause mortality and MACEs was high in patients with ESKD receiving dialysis [8]. Bolignano et al. identified 18 studies and found that the overall PH prevalence was 33% in any stage CKD, and was associated with an elevated risk of all-cause mortality, cardiac death, and non-fatal MACEs [14]. Furthermore, PH was demonstrated as a risk factor for all-cause mortality and cardiac death in the general population [85]. Thus, PH management is associated with improved prognosis, and the symptoms of dyspnea, fatigue, chest pain, and systemic edema in patients with CKD should be cautiously screened. Nowadays, the noninvasive Doppler echocardiography is widely used to rapidly diagnose PH [86].

Although subgroup analyses found PH associated with an increased risk of all-cause mortality in all subgroups of patients with CKD, we noted the association of PH with the risk of all-cause mortality might differ when stratified by disease status and history of CVD. Patients with ESKD showed and increased risk of all-cause mortality than patients with earlier stage CKD or renal transplant. A possible explanation could be that ESKD is the most debilitating stage with patients requiring renal replacement therapy [87–89]. Moreover, the comorbidities, albumin, hemoglobin, and vascular access could explain the high rate of mortality [90]. The survival rate in patients with ESKD on dialysis can be affected by dialysis per session, hypertension, and infection [91]. Finally, CVD history reflects patients’ disease status, and the recurrence of CVD was higher, which is associated with an elevated risk of all-cause mortality. Therefore, these results should be further evaluated according to individuals’ characteristics in a large-scale prospective study.

The results of subgroup analyses were consistent with overall analysis for the relationship between PH and MACEs in patients with CKD, while the strengths of associations varied by country. We noted that MACEs related to PH were more evident in pooled retrospective studies, studies performed in Eastern countries, mean age ≥60.0 years, CVD proportion < 60.0%, hypertension proportion <50.0%, and in patients with ESKD. The potential reasons for these were: (1) The study design may affect the evidence level, and the pooled conclusion may be affected by inevitable selection and recall biases; (2) The proportion of PH and disease management of patients with CKD differs between Eastern and Western countries, affecting the strength of association of PH with the risk of MACEs in patients with CKD; (3) The prevalence of MACEs in older patients was higher than that in young patients [92]; (4) Interestingly, the risk of MACEs related to PH in patients with CKD was higher for individuals at low risk of MACEs (CVD proportion < 30.0%, hypertension proportion < 50.0%), which can be explained by background therapies and requires further evaluation; and (5) renal function could affect atherosclerosis and clearance of metabolic waste products in the body, which is crucial in patients with ESKD [93,94]. Finally, the association of PH with cardiac death in CKD patients did not differ according to predefined factors, which could be due to smaller number of included studies reporting such associations, and insufficient power to detect potential significant associations.

## Strength and limitations

The strengths of this study were: (1) the analysis based on 50 studies, and the results of this study were robust than any individual study; (2) the study comprehensive reported the prevalence, and prognostic role of PH in CKD patients; (3) the stability of summary results was assessed using sensitivity analyses *via* sequential removal of single studies; and (4) stratified analyses were performed for the prevalence, and prognostic role of PH, which helps determine patients with CKD at high risk of PH, and improve the prognosis of PH.

Nonetheless, several shortcomings of this study should be acknowledged. First, both prospective and retrospective studies were included, thus, the results could be biased by selection and confounding factors. Second, the heterogeneity across included studies was not fully explained by sensitivity and subgroup analyses, which could attribute to varied disease status, treatment strategies, and morbidity. Third, the definition of PH was not consistent, which could affect the prevalence of PH. Fourth, the prognosis of CKD patients may be affected by the severity of PH, which was not addressed in our study. Fifth, this study is based on published articles and does not include unpublished data, hence publication bias is inevitable. Furthermore, differences in the results of Egger and Begg analyses mainly stem from their reliance on distinct statistical principles, each having differing sensitivities and specificities. Consequently, in practice, both Egger and Begg tests are employed concurrently. Finally, considering the analysis based on pooled data, and individual data was not available, which restricted us conducted more detailed analyses.

## Conclusion

In conclusion, this study found that overall PH prevalence patients with any stage CKD was 38%, while that in specific CKD stage was higher in patients with ESKD (predialysis) (39%) and ESKD (HD) (42%). Moreover, we identified predictors for PH in patients with CKD, which can help screen for patients at high risk of PH. Furthermore, we noted that PH in patients with CKD was associated with an increased risk of all-cause mortality, MACEs, and cardiac death. Finally, we noted that the relationship between PH and all-cause mortality may be affected by CVD history, while MACEs related to PH might differ when stratified by country. Therefore, further large-scale prospective studies are required to evaluate the prognosis of CKD related to the magnitude of mean PAP.

## Supplementary Material

Supplemental Material
