# Supplementary material for: Predictors, prevalence and prognostic role of pulmonary hypertension in patients with chronic kidney disease: a systematic review and meta-analysis
Source: Ren Fail. 2024 Jun 28;46(2):2368082. doi: 10.1080/0886022X.2024.2368082 (PMC11216249; doi:10.1080/0886022X.2024.2368082)
Supplement: Supplemental Material [file IRNF_A_2368082_SM6036.zip › Supplementary file 1.docx]

**Search strategy in PubMed:**

#1. “Chronic Kidney Disease*" OR "Chronic Kidney disorder" OR "Chronic Kidney Insufficiencies" OR "Chronic Kidney Insufficiency" OR "Chronic nephropathy" OR "Chronic Renal Disease*" OR "Chronic Renal failure" OR "Chronic Renal Insufficiencies" OR "Chronic Renal Insufficiency" OR "Kidney chronic failure" OR "Kidney failure, chronic" OR "Kidney function, chronic disease" OR "Chronic Kidney Failure" OR "Chronic Renal Failure" OR "End Stage Kidney Disease" OR "End stage kidney failure" OR "End Stage Renal Disease" OR "End stage renal dysfunction" OR "End Stage Renal Failure" OR "End stage renal impairment" OR "End stage renal insufficiency" OR "ESRD" OR "Stage 5 kidney disease" OR "Stage 5 renal disease" OR "Kidney Failure*" OR "Kidney Insufficiencies" OR "Kidney Insufficiency" OR "maternal kidney failure" OR "Renal Failure*" OR "Renal Insufficiencies" OR "renal insufficiency" OR "terminal kidney failure" OR "acute dialysis" OR "coil dialysis" OR "Dialyses" OR "Dialyses, Extracorporeal" OR "Dialysis, Extracorporeal" OR "Dialysis, Renal" OR "flow dialysis" OR "Hemodialyses" OR "Hemodialysis" OR "Renal Dialyses"

#2. "Renal Insufficiency, Chronic" OR "Kidney Failure, Chronic" OR "Renal Insufficiency" OR "Renal Dialysis"

#3. "Pulmonary Hypertension" OR "essential pulmonary hypertension" OR "familial primary pulmonary hypertension" OR "hypertensive pulmonary vascular disease" OR "idiopathic pulmonary arterial hypertension" OR "lung arterial hypertension" OR "lung artery hypertension" OR "lung hypertension" OR "primary pulmonary hypertension" OR "pulmonary arterial hypertension" OR "pulmonary artery hypertension" OR "pulmonary fixed hypertension" OR "pulmonary hypertensive disease*" OR "pulmonary hypertensive disorder*"

#4. Pulmonary Hypertension

#5. (#1 OR #2) AND (#3 OR #4)

**Search strategy in EmBase:**

#1. ('Chronic Kidney Disease*' OR 'Chronic Kidney disorder' OR 'Chronic Kidney Insufficiencies' OR 'Chronic Kidney Insufficiency' OR 'Chronic nephropathy' OR 'Chronic Renal Disease*' OR 'Chronic Renal failure' OR 'Chronic Renal Insufficiencies' OR 'Chronic Renal Insufficiency' OR 'Kidney chronic failure' OR 'Kidney failure, chronic' OR 'Kidney function, chronic disease' OR 'Chronic Kidney Failure' OR 'Chronic Renal Failure' OR 'End Stage Kidney Disease' OR 'End stage kidney failure' OR 'End Stage Renal Disease' OR 'End stage renal dysfunction' OR 'End Stage Renal Failure' OR 'End stage renal impairment' OR 'End stage renal insufficiency' OR 'ESRD' OR 'Stage 5 kidney disease' OR 'Stage 5 renal disease' OR 'Kidney Failure*' OR 'Kidney Insufficiencies' OR 'Kidney Insufficiency' OR 'maternal kidney failure' OR 'Renal Failure*' OR 'Renal Insufficiencies' OR 'renal insufficiency' OR 'terminal kidney failure' OR 'acute dialysis' OR 'coil dialysis' OR 'Dialyses' OR 'Dialyses, Extracorporeal' OR 'Dialysis, Extracorporeal' OR 'Dialysis, Renal' OR 'flow dialysis' OR 'Hemodialyses' OR 'Hemodialysis' OR 'Renal Dialyses'):ti,ab,kw

#2. (‘Chronic kidney failure' or 'End-stage renal disease' or 'kidney failure' or 'dialysis')/exp

#3. ('Pulmonary Hypertension' OR 'essential pulmonary hypertension' OR 'familial primary pulmonary hypertension' OR 'hypertensive pulmonary vascular disease' OR 'idiopathic pulmonary arterial hypertension' OR 'lung arterial hypertension' OR 'lung artery hypertension' OR 'lung hypertension' OR 'primary pulmonary hypertension' OR 'pulmonary arterial hypertension' OR 'pulmonary artery hypertension' OR 'pulmonary fixed hypertension' OR 'pulmonary hypertensive disease*' OR 'pulmonary hypertensive disorder*'):ti,ab,kw

#4. 'Pulmonary Hypertension'/exp

#5. (#1 OR #2) AND (#3 OR #4)

**Search strategy in Cochrane:**

#1. ('Chronic Kidney Disease*' OR 'Chronic Kidney disorder' OR 'Chronic Kidney Insufficiencies' OR 'Chronic Kidney Insufficiency' OR 'Chronic nephropathy' OR 'Chronic Renal Disease*' OR 'Chronic Renal failure' OR 'Chronic Renal Insufficiencies' OR 'Chronic Renal Insufficiency' OR 'Kidney chronic failure' OR 'Kidney failure, chronic' OR 'Kidney function, chronic disease' OR 'Chronic Kidney Failure' OR 'Chronic Renal Failure' OR 'End Stage Kidney Disease' OR 'End stage kidney failure' OR 'End Stage Renal Disease' OR 'End stage renal dysfunction' OR 'End Stage Renal Failure' OR 'End stage renal impairment' OR 'End stage renal insufficiency' OR 'ESRD' OR 'Stage 5 kidney disease' OR 'Stage 5 renal disease' OR 'Kidney Failure*' OR 'Kidney Insufficiencies' OR 'Kidney Insufficiency' OR 'maternal kidney failure' OR 'Renal Failure*' OR 'Renal Insufficiencies' OR 'renal insufficiency' OR 'terminal kidney failure' OR 'acute dialysis' OR 'coil dialysis' OR 'Dialyses' OR 'Dialyses, Extracorporeal' OR 'Dialysis, Extracorporeal' OR 'Dialysis, Renal' OR 'flow dialysis' OR 'Hemodialyses' OR 'Hemodialysis' OR 'Renal Dialyses'):ti,ab,kw

#2. ‘Renal Insufficiency, Chronic' or 'Kidney Failure, Chronic' or 'Renal Insufficiency' or 'dialysis'

#3. ('Pulmonary Hypertension' OR 'essential pulmonary hypertension' OR 'familial primary pulmonary hypertension' OR 'hypertensive pulmonary vascular disease' OR 'idiopathic pulmonary arterial hypertension' OR 'lung arterial hypertension' OR 'lung artery hypertension' OR 'lung hypertension' OR 'primary pulmonary hypertension' OR 'pulmonary arterial hypertension' OR 'pulmonary artery hypertension' OR 'pulmonary fixed hypertension' OR 'pulmonary hypertensive disease*' OR 'pulmonary hypertensive disorder*'):ti,ab,kw

#4. 'Pulmonary Hypertension'

#5. (#1 OR #2) AND (#3 OR #4)
