# Supplementary figures and images for: Predictors, prevalence and prognostic role of pulmonary hypertension in patients with chronic kidney disease: a systematic review and meta-analysis
Source: Ren Fail. 2024 Jun 28;46(2):2368082. doi: 10.1080/0886022X.2024.2368082 (PMC11216249; doi:10.1080/0886022X.2024.2368082)

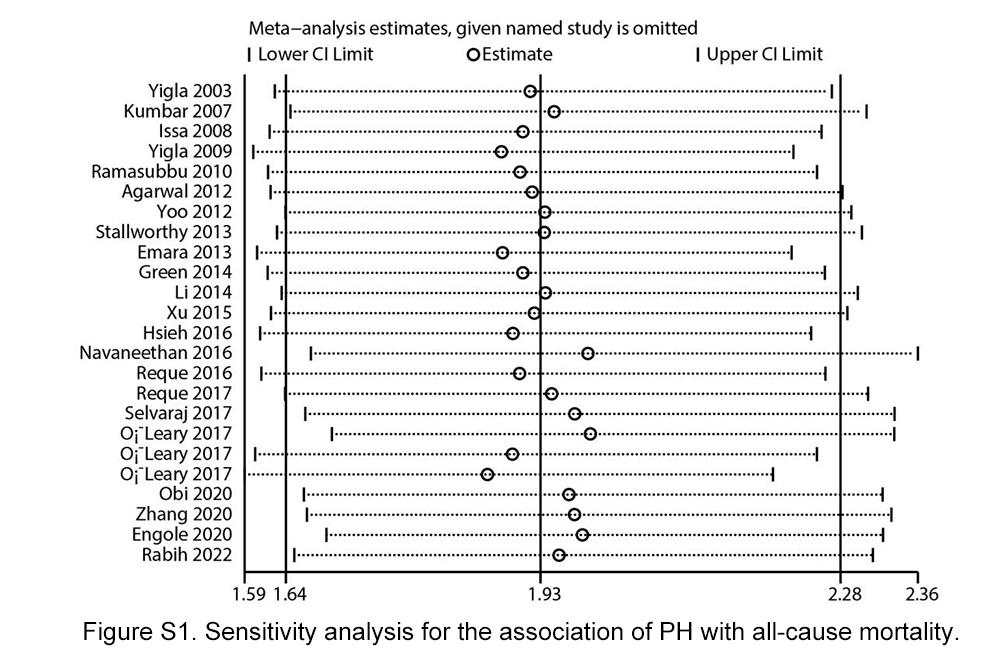

Supplement: Supplemental Material [file IRNF_A_2368082_SM6036.zip › Figure S1.tif]

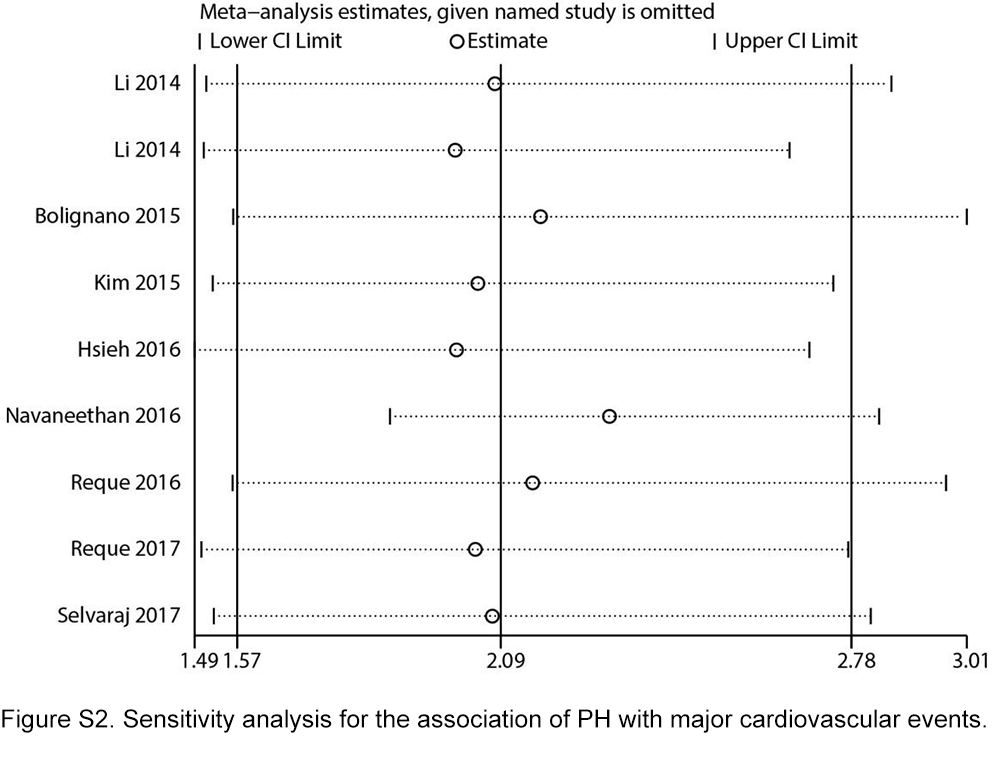

Supplement: Supplemental Material [file IRNF_A_2368082_SM6036.zip › Figure S2.tif]

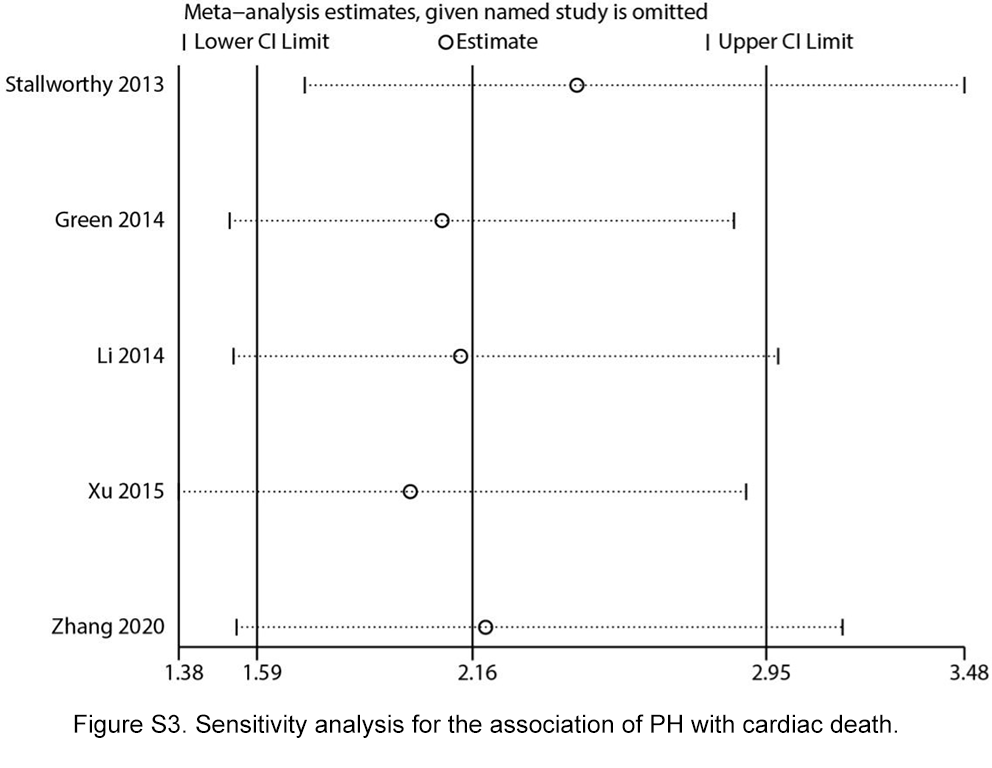

Supplement: Supplemental Material [file IRNF_A_2368082_SM6036.zip › Figure S3.tif]
